# Comparative analysis of oral saliva microbiomes and metabolites in Han population at different altitudes

**DOI:** 10.3389/fmicb.2024.1468365

**Published:** 2024-11-13

**Authors:** Junping Li, Lamu Suonan, Jiangsong Lin, Jiangcuo Zhaxi, Ming Gong, Jian Li, Dawa Langjie, Lulu Zhu, Qiangjiu Shique, Cheng Chen

**Affiliations:** ^1^Key laboratory of Shaanxi Province for Craniofacial Precision Medicine Research, College of Stomatology, Xi’an Jiaotong University, Xi’an, China; ^2^Department of Emergency, College of Stomatology, Xi’an Jiaotong University, Xi’an, China; ^3^Department of General Dentistry, College of Stomatology, Xi’an Jiaotong University, Xi’an, China; ^4^Ali Regional People’s Hospital, Tibet, China

**Keywords:** high-altitude, microbiomes, metabolites, 16S rRNA gene sequencing, non-targeted metabolomics, saliva

## Abstract

**Objective:**

This study investigated the differences in oral saliva microbiota composition and metabolic products among Han Chinese populations living at different altitudes, as well as their correlations.

**Method:**

The analysis was conducted using the 16S rRNA gene sequencing method and untargeted metabolomics.

**Results:**

16S gene sequencing results showed significant differences in bacterial diversity and composition between HH (High altitude Han) group and LH (Low altitude Han) group. LEfSe analysis showed that Selenomonas, Leptotrichia, Veillonella, Prevotella relatively abundant are higher in HH group, Haemophilus, Neisseria, Actinobacillus, Aggregatibacter are higher in LH group (*p*<0.05). Furthermore, as depicted in the phylogenetic tree, there are differences observed between the two groups at all taxonomic levels: 4 phyla, 6 classes, 6 orders, 9 families, 9 genera and 8 species (*p*<0.05). After conducting PICRUSt functional prediction analysis, we identified 11 significantly different KEGG categories (level 2) between the two groups. These categories primarily encompass energy metabolism, amino acid metabolism, and carbohydrate metabolism. Furthermore, non-targeted metabolomics analysis revealed a total of 997 distinct metabolites in the two groups. These differentiated metabolites can be classified into 13 Class I categories including amino acids and their metabolites, benzene and its derivatives, organic acids and their derivatives, heterocyclic compounds, aldehydes, ketones and esters, nucleotides and their metabolites among others. Additionally, fatty acyl compounds, alcohols and amines as well as glycerophospholipids are present along with carbohydrates and other physiologically active components such as hormones. Finally, Pearson correlation analysis of the top 20 differential metabolites with microorganisms demonstrated an interaction between them; however further experimental verification is required to elucidate the specific mechanism of action.

**Conclusion:**

Therefore, this study revealed the effect of altitude on oral saliva microbes and metabolites, as well as their correlations.

## 1 Introduction

Due to the characteristics of low oxygen, low atmospheric pressure, high altitude and high ultraviolet radiation, high-altitude areas are considered to be one of the most extreme environments on Earth, and also one of the places which are not suitable for human survival (Zhou et al., 2023). Compared with low-altitude areas, animals and plants in high-altitude extreme environments, including human metabolism, have significant changes in their physiology and phenotypes, which can also have a significant impact on individual health (AlShahrani et al., 2020). Oral micro-environment is very important for long-term oral health, and it is very easy to be affected by the external environment. At extremely high-altitude areas, changes in ultraviolet rays, atmospheric pressure, temperature, and oxygen content may have a great impact on the oral micro-environment, which in turn affects oral health (Liu et al., 2021). According to the survey, the incidence of oral caries and periodontal disease in extremely high-altitude areas is significantly higher than that in low-altitude areas (Dalmády et al., 2020; Guan et al., 2020). This may be closely related to the changes in the oral micro-environment.

In addition, research has confirmed that local people who have lived at extremely high-altitudes for generations have some changes in their genes to combat the effects of the environment, making them more resistant to hypoxia and the effects of all kinds of harsh environments on their bodies (Dong et al., 2021). But their average life expectancy is still lower than at lower altitudes (Lorenzo et al., 2014). Therefore, it is necessary to study the effects of extreme environments on living organisms.

Due to its rich contents, saliva plays a very positive role in the health of the body and the oral cavity. The changes of its content and structure also indirectly reflect the health of the mouth and the whole body (Czesnikiewicz-Guzik and Górska, 2020). Previous studies have found that hypoxia, strong ultraviolet radiation and cold can affect the structure of gut and skin microbiota in high-altitude people (Liu et al., 2021). It is well known that the structure of oral microbial flora has an important impact on oral health, and the imbalance of oral microbial structure may cause oral diseases (Bourgeois et al., 2019). A few studies have found changes in the structure of microbes in the oral cavity as oxygen content gradually decreases with elevation (Xiao et al., 2020). After excluding the influence of genetic factors, what are the changes in oral microbial structure and salivary metabolites of individuals at extremely high altitude compared with those at low altitude? Are all characteristic metabolites present? Is there any effect on the development and progression of systemic and oral diseases? What are the links between the oral micro-environment and saliva metabolism? This study focuses on these issues.

Microbes produce metabolites through metabolic activities, and metabolites can influence microbial growth and metabolic processes (Kuramitsu et al., 2007). Salivary metabolites are involved in multiple cellular functions, such as direct regulation of gene expression, as well as effectors of molecular events leading to disease (Nielsen, 2017). The main source of salivary metabolites is the products of the oral metabolic pathway and also contains salivary metabolites produced by microorganisms (Gardner et al., 2018; Gardner et al., 2019). This study by detecting high-altitude populations of oral saliva microbial structure and the change of the metabolites of saliva, comparative analysis and ordinary high-altitude difference between oral microenvironment and saliva metabolism, and analyze the individual baseline of saliva and metabolites of microorganisms and the relationship between environment and metabolism. In addition, through this study, some characteristic metabolites and microbial changes in saliva metabolism at extremely high-altitude were screened, which provided basic data for subsequent scholars.

## 2 Materials and methods

### 2.1 Ethical statement

In accordance with the ethical guidelines of the Declaration of Helsinki, the Ethics Committee of Xi’an Jiaotong University Stomatological Hospital and Ali District People’s Hospital approved the experimental protocol, and the written informed consent was obtained, and the privacy of the participants was always protected.

### 2.2 Sample collection

Nine long-term Han residents (more than 20 years, non-generational residence) at extremely high-altitude in Ali, Tibet, and ten long-term Han residents at low-altitude were included in this study. All participants signed informed consent. No antibiotics were used within 1 month before sampling, and fasting was started 10 h before sampling. The saliva is accumulated in the oral cavity during the resting state and subsequently spitting into the sterile centrifuge tube, resulting in a total volume of 5 mL. After centrifugation, the saliva was stored in a 2 mL cryogenic tube and stored at−80°C.

The exclusion criteria encompass individuals who have undergone dental treatment in the past 6 months, those with severe oral diseases (such as periodontitis, oral mucosal diseases, and oral cancer), systemic diseases, pregnant or lactating women, changes in diet, and a history of smoking more than 100 cigarettes (Yu et al., 2017).

### 2.3 DNA extraction, polymerase chain reaction (PCR), and Illumina NovaSeq sequencing

Saliva samples were sent to Wuhan Maiwei Metabolism Biotechnology Co., Ltd. (Wuhan, China), and microbial DNA was extracted, amplified and sequenced according to standard procedures. The V4-V5 region of 16S rRNA was amplified with forward primer 515F (5′-GTGCCAGCMGCCGCGGTAA-3′) and reverse primer 806R (5′-GGACTACHVGGGTWTCTAAT-3′). The PCR products were tested by electrophoresis using 2% agarose gel; the qualified PCR products were purified by magnetic beads, quantified by the PCR product, 2% agarose gel to detect the PCR products, and the glue recovery kit provided by Qiagen, co td. Library construction was performed using the TruSeq® DNA PCR-Free Sample Preparation Kit library building kit, and the constructed libraries were quantified by Qubit and Q-PCR, before computer sequencing using NovaSeq6000.

### 2.4 Non-targeted metabolomics study

#### 2.4.1 Sample extraction

The sample stored at −80°C refrigerator was thawed on ice and vortexed for 10 s. A 150 μL extract solution (ACN: Methanol = 1:4, V/V) containing internal standard was added into 50 μL sample. Then the sample was vortex for 3 min and centrifuged at 12000 rpm for 10 min (4°C). A 150 μL aliquots of the supernatant was colleted and placed in −20°C for 30 min, and then centrifuged at 12000 rpm for 3 min (4°C). 120 μL aliquots of supernatant were transferred for LC–MS (Liquid chromatography-mass spectrometry) analysis.

#### 2.4.2 Acquisition conditions for chromatmass spectrum

##### 2.4.2.1 C18 chromatographic conditions

(1) Chroma column: Waters ACQUITY UPLC BEH C18 1.8 μm, 2.1 mm × 100 mm. (2) Mobile phase A: ultrapure water (0.1% formic acid); mobile phase B: acetonitrile (0.1% formic acid) (3) Column temperature of the instrument: 40°C; flow rate: 0.40 mL/min; sample quantity: 2uL.

##### 2.4.2.2 Agilent 6,545 QTOF mass spectrum conditions

Agilent 6545 QTOF, mass spectrum conditions (Table 1)

**Table 1:** Agilent 6,545 QTOF, mass spectrum conditions.

	ESI+	ESI−
Voltage (V)	2,500	1,500
Gas flow (L/min)	8	8
Fragmetor (V)	135	135
Gas temperature (°C)	325	325
Sheath temperature (°C)	325	325
Sheath flow (L/min)	11	11
Nebulizer (V)	40	40

### 2.5 Data processing

#### 2.5.1 16S sequencing data processing

Each sample data was split from the lower machine data based on the Barcode sequence and the PCR amplification primer sequence, and the Barcode and primer sequences were amputated. The raw reads were filtered using fastp to obtain high quality reads by: automatically detect and remove adaptor sequence; remove reads with 15 or more *N* bases; remove reads (mass ≤ 20) over 50%; delete reads with average mass below 20 in the 4-base window; remove polyG in the tail; remove reads longer than 150 bp. High-quality double-end reads were then stitched together using FLASH to obtain high-quality Tags data. Tags sequences were detected by alignment of vsearch to the species annotation database, and the chimera sequences were finally removed to obtain the final valid data.

#### 2.5.2 Preprocessing of metabolomics data

The original data file acquisited by LC–MS was converted into mzML format by ProteoWizard software. Peak extraction, peak alignment and retention time correction were, respectively, performed by XCMS program. The “SVR” (Support Vector Regression) method was used to correct the peak area. The peaks with detetion rate lower than 50% in each group of samples were discarded. After that, metabolic identification information was obtained by searching the laboratory’s self-built database, integrated public database, AI database and metDNA.

## 3 Results

### 3.1 Taxonomic analysis

**Figure 1:**
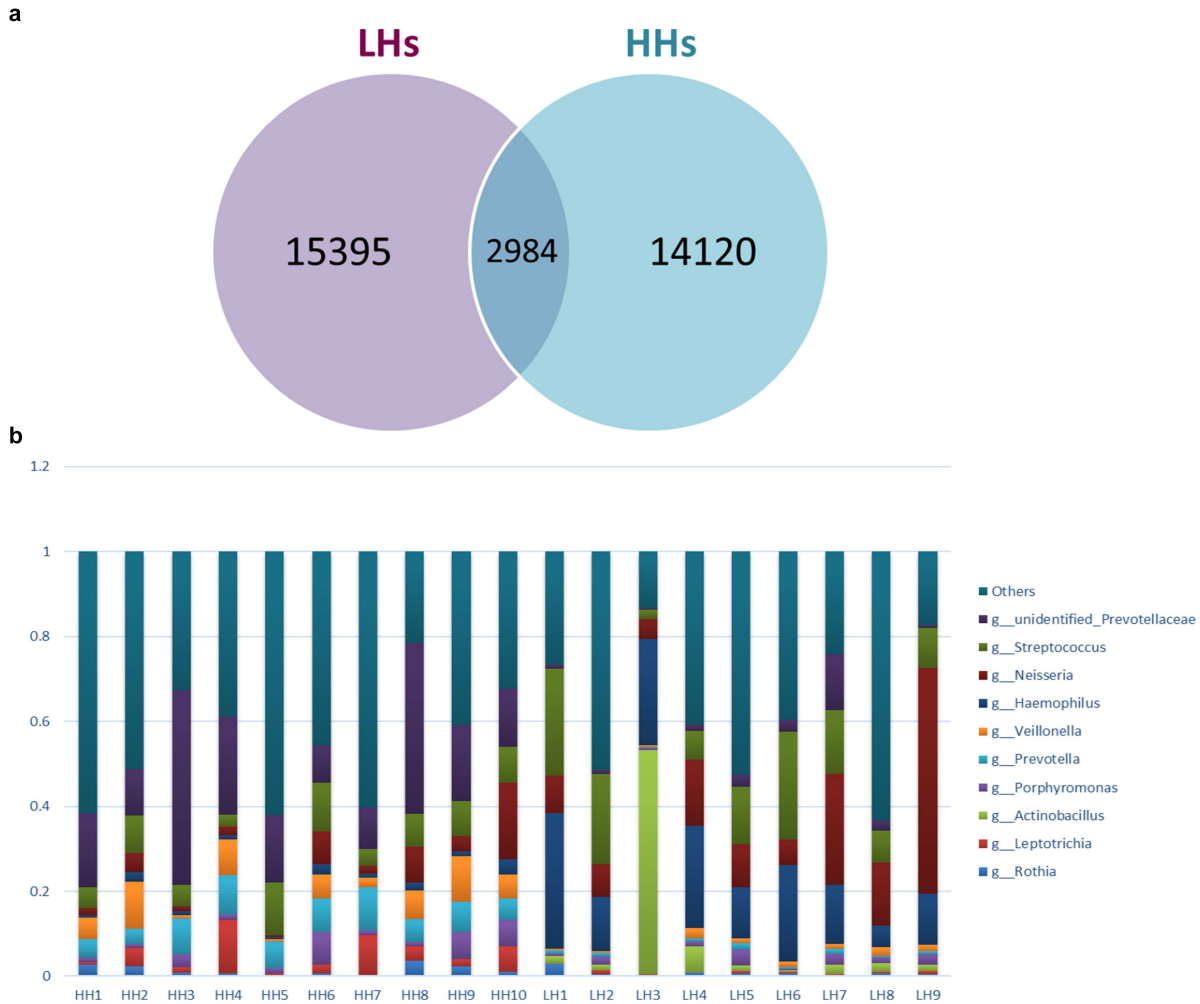
(a) Venn graph. LH, Low altitude Han population; HH, High altitude Han population. (b) Genus level Histogram of relative abundance of species. The horizontal coordinate is the sample name, the vertical coordinate is the relative abundance, and the top 10 genera with the highest abundance in both groups are: unidentified *Prevotellaceae, Streptococcus, Neisseria, Haemophilus, Veillonella, Prevotella, Porphyromonas, Actinobacillus, Leptotrichia* and *Rothia*.

Deblur algorithm was used for denoising analysis, by comparing the Hamming distances between sequences within a sample and between samples with the upper error curve. Combined with greedy algorithm, it obtains Amplicon Sequence Variants (ASVs) as variants of amplification sub-sequences, enabling the analysis of shared and unique ASVs between two groups of samples, and generating a Venn diagram (Figure 1a). The Venn diagram displayed the composition of species: 14,120 ASVs were detected in the HH group, 15,395 ASVs were detected in the LH group, with an overlap of 2,984 ASVs. Based on species annotation results, the top 10 abundant species at Phylum, Class, Order, Family, Genus and Species levels were selected in each sample to generate a stacked bar plot showing their relative abundances (Figure 1b).

### 3.2 Beta diversity analysis

**Figure 2:**
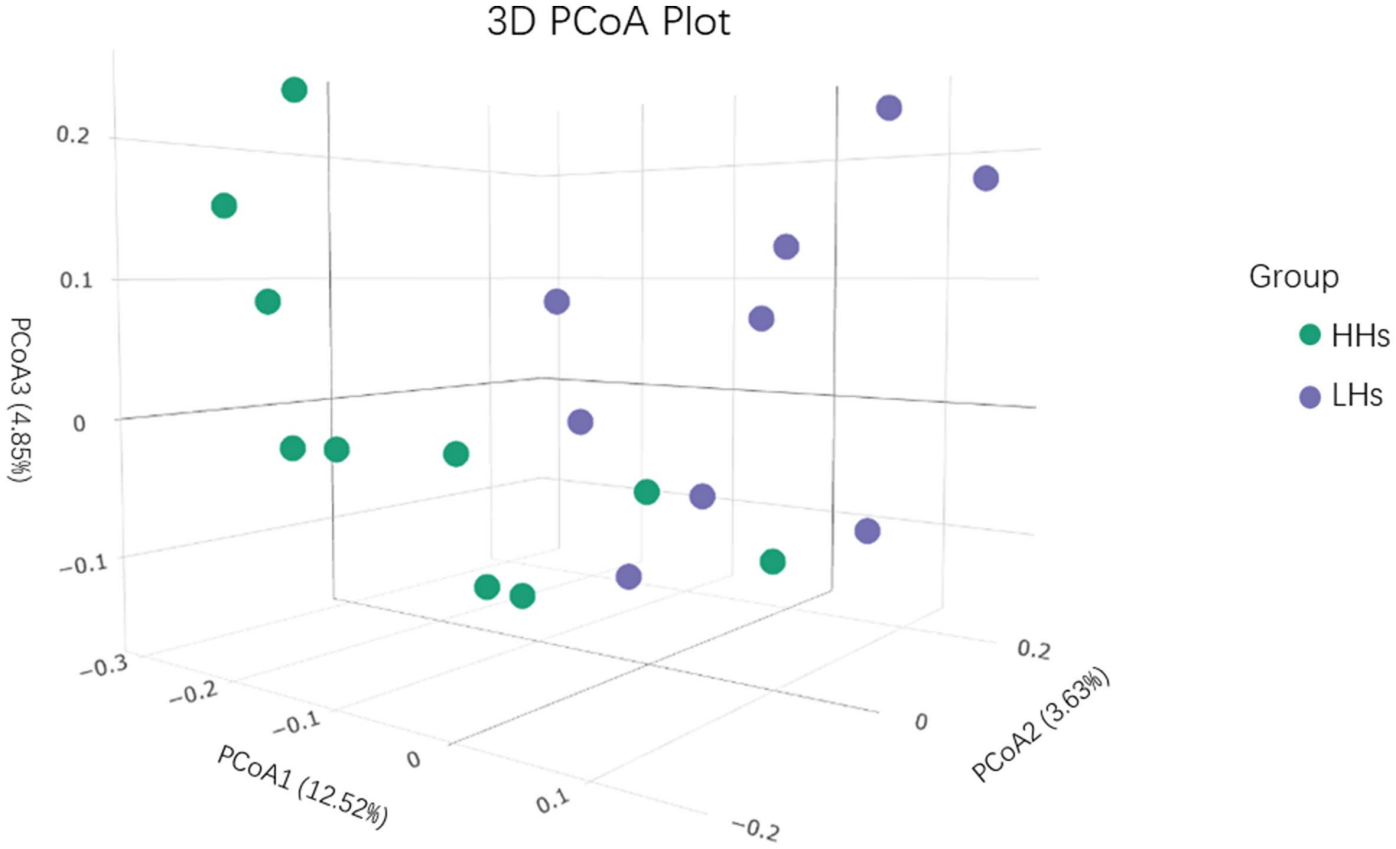
PCoA 3Dplot. Principal Coordinate Analysis (PCoA) based on Weighted Unifrac distance.

There was no significant difference in alpha diversity between the two groups, however, beta diversity analysis revealed a noticeable difference between LH group and HH group (*p*<0.05). We conducted Principal Coordinate Analysis (PCoA) based on Weighted Unifrac distance to further investigate whether there are differences in the salivary microbiota structure between the HH group and LH group (Figure 2). The distribution of the two groups overlapped partially, but there were also obvious separations. PC1 explained 12.52% of the variation observed. PC2 and PC3 explained 3.63 and 4.85% of the variation, respectively.

### 3.3 Significant differential microflora analysis by LEfSe

**Figure 3:**
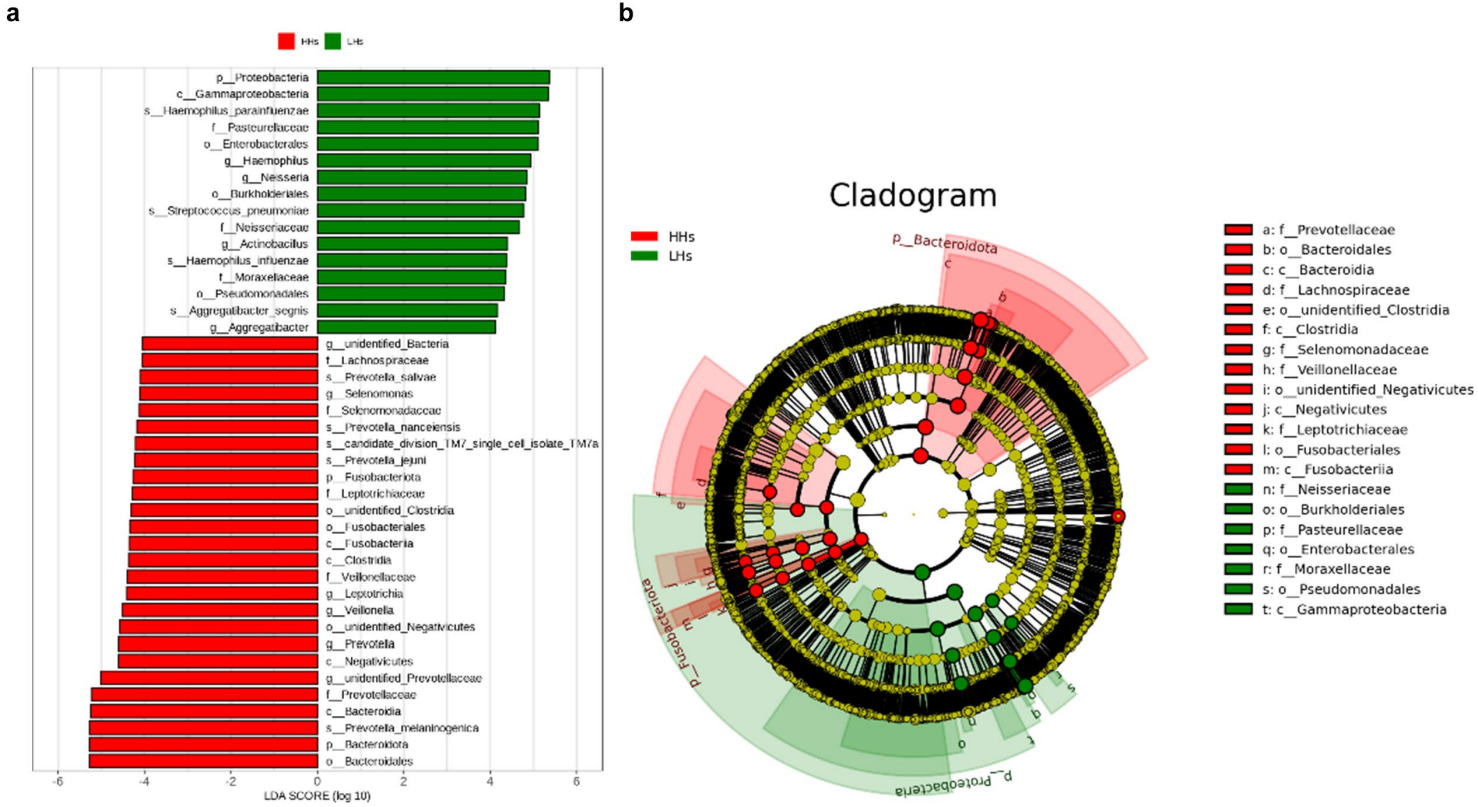
(a) The LDA value bar chart. LefSe showed a list of specific saliva microbiota that enable discrimination between the LH and HH group. Taxa enriched in LH group are indicated with a positive LDA score (green), and taxa enriched in HH group have a negative score (red). Only taxa meeting an LDA significant threshold of 4 are shown. For taxa, which were defined as unclassified or Incertae-Sedis, the name of a higher taxon level was added before its taxon abbreviation. p, phylum; c, class; o, order; f, family; g, genus; s, species. (b) The dominant taxa between LH group and HH group were analyzed using LEfSe. Visualization of differential taxa on a phylogenetic tree from phylum to genus level. In an evolutionary cladistic diagram, the concentric circles represent taxonomic levels ranging from phylum to genus (or species). Each small circle at a specific classification level signifies a classification within that level, with the diameter of the circle being proportional to its relative abundance. Color coding principle: Uniform yellow color is assigned to species with no significant differences. Red nodes denote important microbial groups in HH group, whereas green nodes indicate important microbial groups in LH group.

To further clarify the differences in saliva microbiota between HH and LH, we performed linear discriminant analysis LDA and effect size analysis (LEfSe) at the genus level. As shown in Figure 3a, the bar chart of the LDA value distribution displays the species with LDA Score greater than the set point (defaulted to 4) and significant abundance differences between the two groups. Species relatively abundant in high-altitude areas include *Selenomonas, Leptotrichia, Veillonella, Prevotella* (*p*<0.05). On the other hand, species relatively abundant in low-altitude areas include *Haemophilus, Neisseria, Actinobacillus, Aggregatibacter* (*p*<0.05). Furthermore, as depicted in the phylogenetic tree (Figure 3b), there are differences observed between the two groups at all taxonomic levels: 4 phyla, 6 classes, 6 orders, 9 families, 9 genera and 8 species (*p*<0.05).

### 3.4 PICRUSt function prediction analysis

**Figure 4:**
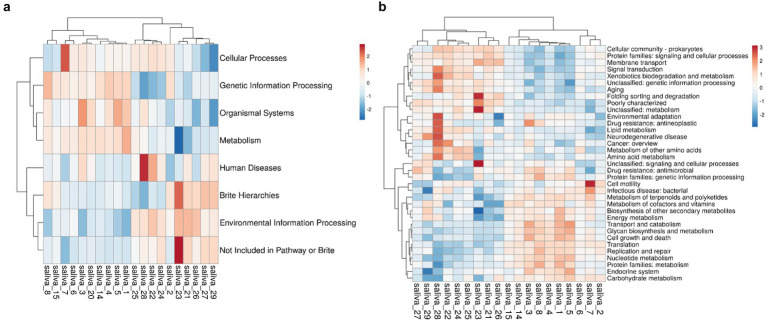
(a) KEGG1 level 1 clustering heat map. (b) KEGG level 2 clustering heat map. The horizontal axis represents the sample, while the vertical axis represents the function. The grid indicates the relative abundance, with higher values depicted in red and lower values in blue. (a) KEGG level 1; (b) KEGG level 2.

We performed metagenomic sequencing analysis of the HH and LH groups to explore whether there were functional differences in the oral saliva microbiota between the two groups. According to the abundance of the database functional annotation in all samples, the top 35 functions and their abundance information in each sample were selected to draw the heatmap, and the heatmap. There are seven categories at the KEGG level 1, including Cellular Processes, Genetic Information Processing, Organismal Systems, Metabolism, Human Diseases, Brite Hierarchies, and Environmental Information Processes (Figure 4a). In order to further clarify which specific function led to the altitude difference between bacteria, we studied 35 KEGG categories (level 2), found 11 KEGG categories between two groups (Figure 4b), these can be divided into three categories: “energy metabolism,” “amino acid metabolism” and “carbohydrate metabolism.”

### 3.5 Analysis of the differential metabolites

**Figure 5:**
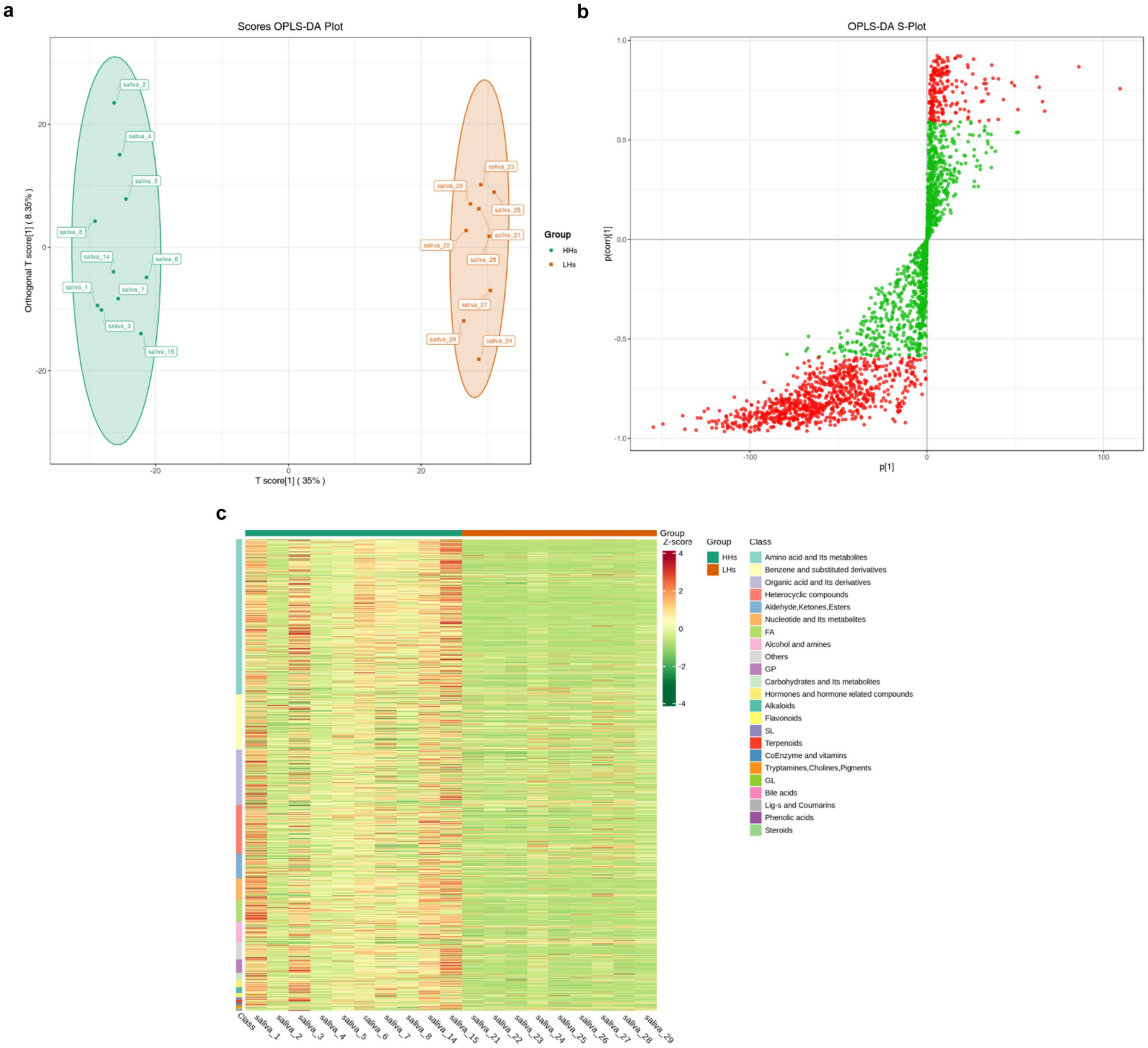
(a) OPLS-DA scorePlot. The horizontal axis represents the difference between groups, while the vertical axis represents the difference within groups. The results indicate a significant difference between the two groups (*p* < 0.01). (b) OPLS-DA S-plot. The horizontal axis represents the covariance between principal components and metabolites, while the vertical axis represents the correlation coefficient between principal components and metabolites. Metabolites closer to the top right corner or bottom left corner indicate more significant differences. The red dots indicate metabolites with VIP values greater than 1, while green dots represent metabolites with VIP values less than or equal to 1. Generally, metabolites with VIP > 1 are considered significantly different. (c) Cluster heat map of differential metabolites. The horizontal axis represents sample information, while the vertical axis represents differential metabolite information. Different colors are used to represent standardized values of relative contents: red indicating high content and green indicating low content.

In order to investigate the metabolic differences between the HH group and LH group, a non-targeted metabolomics analysis was conducted, followed by orthogonal partial least squares discriminant analysis (OPLS-DA) to generate the OPLS-DA score plot (Figure 5a) and S-plot plot of OPLS-DA (Figure 5b). A total of 997 metabolites were found to exhibit significant differences between the two groups (*p* < 0.05). These metabolites were primarily classified into 13 categories according to Class I classification, including amino acids and their derivatives, benzene and its derivatives, organic acids and their derivatives, heterocyclic compounds, aldehydes, ketones, ester compounds, etc. Additionally identified were nucleotides and their derivatives, fatty acyl compounds, alcohols and amines as well as glycerol phospholipids. Carbohydrates and their derivatives along with hormones/hormone-related compounds constituted another category while alkaloids represented an additional group. Furthermore, unclassified compounds were also observed. Subsequently employing the ComplexHeatmap package in R software facilitated generation of a cluster heat map (Figure 5c), which revealed significantly higher levels of metabolite content in the HH group compared to that in the LH group thereby indicating heightened metabolic activity among individuals residing at high altitudes.

### 3.6 Pearson correlation analysis of differential microorganisms and differential metabolites

**Figure 6:**
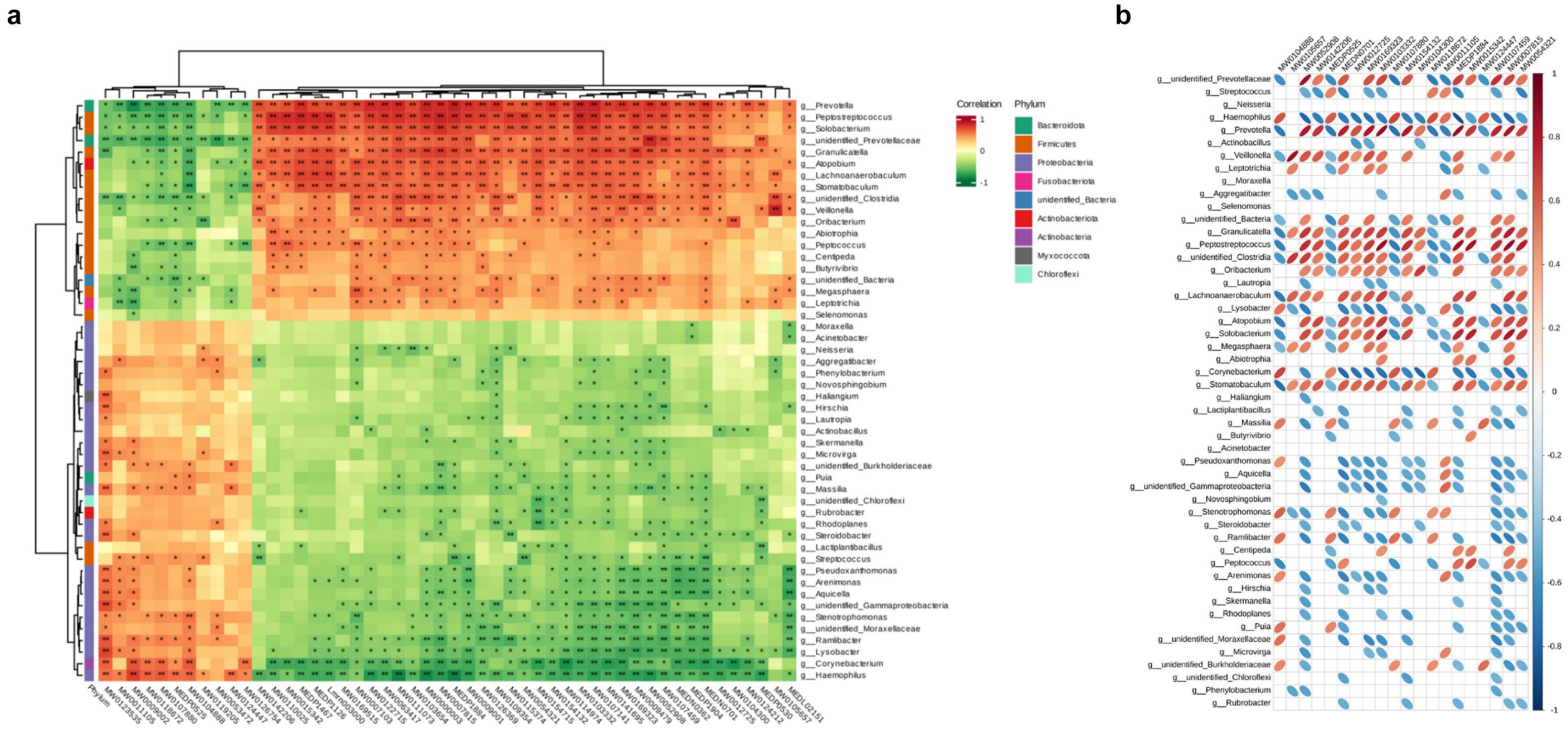
Hierarchical clustering heat map of Pearson correlations between differential microorganisms and differential metabolites in two groups. The metabolite numbers are shown below (see Supplementary Table 1), with the microorganisms indicated on the right. The evolutionary tree on the left represents hierarchical clustering results for microorganisms, while the tree above represents hierarchical clustering results for metabolites. Red indicates positive correlation, while green indicates negative correlation. Significance testing of correlation coefficients showed that *p* < 0.05 is considered significantly different and denoted by “*”, and *p* < 0.01 is considered highly significant and denoted by “**”. (b) Heatmap of Pearson correlation between TOP20 differential metabolites and differential microorganisms. Microbial communities are represented on the left, while metabolite profiles are displayed at the top. Positive correlations are depicted by red ovals, whereas negative correlations are indicated by blue ovals. The size of the ellipse reflects the magnitude of correlation, with smaller ellipses representing stronger associations. Blank spaces denote non-significant (*p* > 0.05). Refer to Supplementary Table 1 for column details.

After conducting correlation analysis, we calculated the Pearson correlation coefficient between differential microbiota and differential metabolites, and displayed the correlation between differentially abundant microbiota and metabolites at various taxonomic levels using a clustering heatmap (Figures 6a,b). We observed a positive correlation with significant higher abundance (*p* < 0.05) between 2′-deoxyadenosine and bacterial genera such as *Veillonella, Prevotella, Clostridia* in the HH group. It is worth noting that 2′-deoxyadenosine is involved in genetic information transfer in almost all biological cells and has an impact on protein synthesis and polysaccharide metabolism. This finding also suggests that populations living at high altitudes may have more active metabolic activities.

## 4 Discussion

### 4.1 Analysis of microbial diversity in saliva

The factors affecting the structure of the oral microbiome include age, race, diet, oral hygiene habits, living environment, and cultural background, which can be broadly divided into genetic factors and external factors (Mason et al., 2014). There is currently no consensus on the extent and priority of the influence of these factors on the oral microbiome (Fiorillo, 2020). The oral cavity, as the starting point of the digestive tract and in communication with the outside environment, is significantly affected by external factors regarding the structure and composition of its microbiota and metabolites (Gilbert and Stephens, 2018). The extreme environment of high-altitude areas, along with unique dietary and oral hygiene habits, is a crucial reason for the distinctive structure of the oral microbiome in local populations. This study aims to analyze the impact of “altitude factor” on the structure of the oral microbiome and salivary metabolites in healthy Han Chinese populations.

The study found no statistically significant difference in *α*-diversity between the two groups, which differs from previous studies. We attribute this to the small sample size. However, *β*-diversity analysis showed significant differences. Principal Coordinates Analysis (PCoA) based on Weighted Unifrac distances revealed clear differences between the two groups, yet there were also similarities. Venn diagrams indicated an overlap of 2,984 ASVs between the two groups, suggesting similar species composition. Any part of the body can develop a higher frequency, abundance, and stability of microbiota over time, known as the “core microbiome (Hu et al., 2013) “. In our study, the abundance of *Firmicutes* was high in both groups, but higher in the HH group, consistent with the findings of Zhao et al. (2022). Some researchers (Hu et al., 2013) believe that *Firmicutes* can encode enzymes related to energy metabolism, producing various digestive enzymes to decompose different substances, thereby promoting metabolism. This also suggests a higher energy expenditure in high-altitude populations.

Among the top five phyla are *Bacteroidetes*, *Proteobacteria*, *Fusobacteria*, and *Actinobacteria*. *Bacteroidetes*, *Fusobacteria*, and *Actinobacteria* were more abundant in the HH group, while *Proteobacteria* were more abundant in the LH group. Lucking et al. found that a higher abundance of *Bacteroidetes* helps the host maintain normal blood pressure (Lucking et al., 2018). Human blood pressure increases with altitude, and studies have shown that microorganisms participate in blood pressure regulation (Stoltzfus et al., 2020), which confirms our findings. Additionally, it has been shown that the composition and metabolic activity of *Bacteroidetes* are regulated by diet and are associated with high fat and protein intake. Given that the high-altitude diet is primarily high in fat and low in carbohydrates, we suspect that the differences in *Bacteroidetes* between the two groups are related to altitude and dietary habits. Contrary to our results, Zhao et al. (2022) found a decrease in *Bacteroidetes* abundance in high-altitude areas, which they attributed to lower fruit intake (Wu et al., 2020). To address these discrepancies, subsequent studies with larger sample sizes are needed for further validation.

At the genus level, we found nine genera with differences between the two groups. *Rothia*, *Capnocytophaga*, *Porphyromonas*, *Prevotella* and *Veillonella* were more abundant in the HH group. In the LH group, *Actinomyces*, *Haemophilus*, *Neisseria*, and *Streptococcus* were more abundant. This result is similar to the findings of Das et al. (2018). Previous reports (Tian et al., 2018) indicated that both laboratory mice under simulated hypoxia and wild house mice in high-altitude areas showed an increase in *Prevotella* numbers. Zeng et al. (2020) found that *Prevotella* helps humans better adapt to the extreme environment of high altitudes. This is related to *Prevotella* ability to promote the conversion of substances into short-chain fatty acids, produce H_2_S, and increase cerebral blood flow, thereby regulating human blood pressure and pulmonary artery pressure to help the host adapt to low pressure and hypoxic environments (Mishra et al., 2020). The optimal growth temperature for *Streptococcus* is about 37°C, and previous studies have found (Zeng et al., 2020) that *Streptococcus* is very abundant in the intestines of animals and humans in low-altitude areas, consistent with our results.

### 4.2 PICRUSt

We used PICRUSt for functional prediction and found that the two groups exhibited similar microbial functional characteristics, which may be related to the core microbiome we mentioned earlier. The top-ranked KEGG level 2 functions for both groups were: amino acid metabolism, carbohydrate metabolism, protein signaling pathways, and cellular processes, indicating vigorous microbial metabolism. In high-altitude areas, high UV radiation and low oxygen levels can lead to DNA and protein damage, and the functions mentioned above can help reduce biomolecular damage, thus protecting the population living in high-altitude areas (Foll et al., 2014).

### 4.3 Differential metabolites

Salivary metabolites mainly originate from products of oral metabolic pathways, especially those produced by microorganisms (Gardner et al., 2018; Gardner et al., 2019). We conducted untargeted metabolomics analysis on saliva samples from both groups and identified 997 differential metabolites, with amino acids and their metabolites (small peptides) being the most numerous. Amino acids can perform the following functions through metabolism in the human body: (1) synthesize proteins and participate in biological functions; (2) convert into acids, hormones, antibodies, creatine, and other nitrogen-containing substances; (3) transform into carbohydrates and fats as energy storage substances; (4) oxidize into carbon dioxide, water, and urea, producing energy. Additionally, amino acids participate in the formation of enzymes, hormones, neurotransmitters, and some vitamins in the body (Paulusma et al., 2022). In our study, small peptide metabolites were significantly higher in the HH group than in the LH group. Cyclo (His-Pro) (CHP) is a cyclic dipeptide formed by proline and histidine through complementary peptide bonds. CHP is an endogenous cyclic dipeptide, a metabolic product of thyrotropin-releasing hormone hydrolyzed by pyroglutamyl aminopeptidase, widely distributed in mammalian gastrointestinal tract, blood, and other tissues and fluids (Wang et al., 2022). Studies have found (De Masi et al., 2023) that endogenous CHP exhibits neurotransmitter-like activity in animals and can regulate animal body temperature through a dopamine-like mechanism, potentially participating in central system temperature regulation to adapt to environmental changes. In our study, CHP was significantly higher in the HH group than in the LH group, closely related to the need for the body to adjust to the low-temperature environment of high altitudes.

### 4.4 Correlation analysis between differential metabolites and differential microorganisms

The hypoxic environment of high altitudes can lead to insufficient blood perfusion in periodontal tissues, affecting the integrity of periodontal tissues (Karl et al., 2018). Meanwhile, hypoxia can cause reduced saliva secretion, decreased self-cleaning ability, and accelerated growth of anaerobic periodontal bacteria, leading to periodontitis (Yang et al., 2019). Studies have found that the abundance of Prevotella is significantly higher in patients with dry mouth than in healthy individuals (Rusthen et al., 2019). In our study, the metabolite itaconic acid was more abundant in the HH group than in the LH group. Itaconic acid, a product of the tricarboxylic acid cycle, has been proven to have outstanding immunoregulatory functions (Li et al., 2020). Xin et al. (2022) found that cell-permeable itaconic acid derivatives can effectively control periodontal inflammation and alveolar bone loss. Pearson correlation analysis of differential metabolites and microorganisms between the HH and LH groups showed that itaconic acid positively correlated with Prevotella and other genera with higher abundance in the HH group. Therefore, we speculate that itaconic acid, as a metabolite, plays an active role in helping the body adapt to the hypoxic environment of high altitudes.

However, we did not conduct in-depth research on the mechanisms of interaction between specific metabolites and microorganisms. In the future, we need to increase the sample size, incorporate targeted studies, and further validate the interaction relationship between microorganisms and metabolites.

Our study has certain limitations. Firstly, the small sample size makes the study relatively one-sided. Based on these results, we believe that increasing the sample size is necessary. Secondly, we have preliminarily screened differential microorganisms and metabolites in the two groups, and their roles need further exploration.

## 5 Conclusion

In summary, our study based on microbial 16S and non-targeted metabolome detection analysis revealed significant differences in the structure of human oral saliva microbial communities and metabolite composition at varying altitudes. In the high- altitude Han population, we observed higher abundance of *Rotella*, *Leptothrix*, *Porphyromonas*, *Prevotella*, and *Veyonia*. Functional prediction analysis indicated that energy metabolism, amino acid metabolism, and carbohydrate metabolism were prominent characteristics distinguishing the two groups. Furthermore, we explored the correlation between differential metabolites and microbes to establish a relationship between them; however, further validation is required to elucidate the underlying mechanisms responsible for this association. We hope that these findings can provide valuable insights for enhancing oral health in high altitude regions.

## Data Availability

The data presented in the study are deposited in the NCBI repository, accession number: PRJNA1179344 http://www.ncbi.nlm.nih.gov/bioproject/1179344.
