# Supplementary material for: Comparative analysis of oral saliva microbiomes and metabolites in Han population at different altitudes
Source: Front Microbiol. 2024 Nov 13;15:1468365. doi: 10.3389/fmicb.2024.1468365 (PMC11610449; doi:10.3389/fmicb.2024.1468365)
Supplement: Supplementary file 1 [file Table_1.docx]

Supplementary Table 1. Metabolite information

| Index | Compounds | Class I | Class II | Formula |
| --- | --- | --- | --- | --- |
| MW0123535 | delta-Hexanolactone | Aldehyde,Ketones,Esters | Esters | C6H10O2 |
| MW0011105 | Zapa | Organic acid and Its derivatives | Organic acid and Its derivatives | C4H6N2O2S |
| MW0009002 | N-cyclopropyl-11-(3-hydroxy-5-pentylphenoxy)undecanamide | Benzene and substituted derivatives | Phenolics | C25H41NO3 |
| MW0118672 | 2-Methoxy-3,5-dimethylpyrimidine | Heterocyclic compounds | Heterocyclic compounds | C7H10N2O |
| MW0107880 | L-Homophenylalanine | Amino acid and Its metabolites | Amino acids | C10H13NO2 |
| MEDP0525 | N-Acetyl-L-alanine | Amino acid and Its metabolites | Amino acid derivatives | C5H9NO3 |
| MW0104888 | 3-(Imidazol-4-yl)-2-oxopropyl dihydrogen phosphate | Aldehyde,Ketones,Esters | Esters | C6H9N2O5P |
| MW0119205 | Mdppp | Aldehyde,Ketones,Esters | Ketones | C14H17NO3 |
| MW0053472 | (1S,3Z,5R,7R)-3-[(3,4-dihydroxyphenyl)-hydroxy-methylene]-1-[(2S)-2-isopropenyl-5-methyl-hex-4-enyl]-6,6-dimethyl-5,7-bis(3-methylbut-2-enyl)bicyclo[3.3.1]nonane-2,4,9-trione | Benzene and substituted derivatives | Phenolics | C38H50O6 |
| MW0124447 | Isatin | Heterocyclic compounds | Indole and Its derivatives | C8H5NO2 |
| MW0126754 | Theobromine | Nucleotide and Its metabolites | Nucleotide and Its metabolites | C7H8N4O2 |
| MW0142206 | Dromostanolone tetrahydropyranyl ether | Aldehyde,Ketones,Esters | Ketones | C25H40O3 |
| MW0110025 | Triethyl citrate | Aldehyde,Ketones,Esters | Esters | C12H20O7 |
| MW0015342 | Prosta-5,13-dien-1-oic acid, 11-hydroxy-9,15-dioxo-, (5Z,8beta,11alpha,13E)- | Hormones and hormone related compounds | Hormones and hormone related compounds | C20H30O5 |
| MEDP1467 | 5-Methyl-2'-deoxycytidine | Nucleotide and Its metabolites | Nucleotide and Its metabolites | C10H15N3O4 |
| MEDP1126 | D-(-)-alpha-Phenylglycine | Amino acid and Its metabolites | Amino acid derivatives | C8H9NO2 |
| Lmrn003000 | DL-3-Phenyllactic acid | Organic acid and Its derivatives | Organic acid and Its derivatives | C9H10O3 |
| MW0169515 | 2-Acetamido-2-Deoxy-D-Mannopyranose | Carbohydrates and Its metabolites | Sugars | C8H15NO6 |
| MW0007103 | F-Amidine (trifluoroacetate salt) | Heterocyclic compounds | Heterocyclic compounds | C16H20F4N4O4 |
| MW0122715 | Aceclidine | Heterocyclic compounds | Heterocyclic compounds | C9H15NO2 |
| MW0063417 | Salvinorin A | Heterocyclic compounds | Heterocyclic compounds | C23H28O8 |
| MW0111073 | Leukotriene B4 ethanolamide | Alcohol and amines | Amines | C22H37NO4 |
| MW0103654 | Riboprine | Nucleotide and Its metabolites | Nucleotide and Its metabolites | C15H21N5O4 |
| MW0000003 | Cinchonan-3,9-diol, 6'-methoxy-, (8alpha,9R)- | Alkaloids | Alkaloids | C20H24N2O3 |
| MW0007815 | Michler's ketone | Benzene and substituted derivatives | Benzene and substituted derivatives | C17H20N2O |
| MEDP1884 | Prolyl-Histidine | Amino acid and Its metabolites | Small Peptide | C11H16N4O3 |
| MW0009001 | N-Cyclopropyl-11-(2-hexyl-5-hydroxyphenoxy)undecanamide | Benzene and substituted derivatives | Phenolics | C26H43NO3 |
| MW0126369 | Quinacrine | Heterocyclic compounds | Heterocyclic compounds | C23H30ClN3O |
| MW0109354 | Pro-Asp | Amino acid and Its metabolites | Small Peptide | C9H14N2O5 |
| MW0109354 | Pro-Asp | Amino acid and Its metabolites | Small Peptide | C9H14N2O5 |
| MW0054321 | Leukotriene B4-3-aminopropylamide | FA | Oxidized lipids | C23H40N2O3 |
| MW0154715 | Okadaic acid | Organic acid and Its derivatives | Organic acid and Its derivatives | C44H68O13 |
| MW0154132 | SAHA-BPyne | Benzene and substituted derivatives | Benzene and substituted derivatives | C27H31N3O5 |
| MW0114974 | N-Acetylmannosamine | Alcohol and amines | Amines | C8H15NO6 |
| MW0103332 | 2'-Deoxyadenosine | Nucleotide and Its metabolites | Nucleotide and Its metabolites | C10H13N5O3 |
| MW0107141 | Glycyl-L-leucine | Amino acid and Its metabolites | Amino acid derivatives | C8H16N2O3 |
| MW0141695 | Arachidonoyl LPA | GP | LPA | C23H42NO7P |
| MW0169323 | Isoliquiritigenin | Flavonoids | Dihydroflavone | C15H12O4 |
| MW0009479 | Piperidolate | Benzene and substituted derivatives | Benzene and substituted derivatives | C21H25NO2 |
| MW0052908 | Finasteride | Heterocyclic compounds | Heterocyclic compounds | C23H36N2O2 |
| MW0107459 | Ile-Pro-Ile | Amino acid and Its metabolites | Small Peptide | C17H31N3O4 |
| MEDN0362 | LPE(18:1/0:0) | GP | LPE | C23H46NO7P |
| MEDP1904 | LPE(18:2/0:0) | GP | LPE | C23H44NO7P |
| MEDN0701 | Itaconic acid | Organic acid and Its derivatives | Organic acid and Its derivatives | C5H6O4 |
| MEDP0530 | 15-deoxy-δ-12,14-PGJ2 | Hormones and hormone related compounds | Hormones and hormone related compounds | C20H28O3 |
| MEDL02151 | 3'-Adenylic acid | Nucleotide and Its metabolites | Nucleotide and Its metabolites | C10H14N5O7P |
| MW0012725 | 19-Oxoandrostenedione | Hormones and hormone related compounds | Hormones and hormone related compounds | C19H24O3 |
| MW0104300 | 1-Phenyl-1-propanone | Aldehyde,Ketones,Esters | Ketones | C9H10O |
| MW0124212 | Ganciclovir | Heterocyclic compounds | Heterocyclic compounds | C9H13N5O4 |
| MW0105657 | Arginyl-glycyl-aspartic acid | Amino acid and Its metabolites | Small Peptide | C12H22N6O6 |

Supplementary Table 2. Abbreviation

| Abbreviation | Full Spelling |
| --- | --- |
| ASVs | Amplicon Sequence Variants |
| LC-MS | Liquid chromatography-mass spectrometry |
| SVR | Support vector regression |
| PCoA | Principal Coordinate Analysis |
| OPLS-DA | Orthogonal partial least squares discriminant analysis |
